# Genomic identification and characterization of *Streptococcus oralis* group that causes intraamniotic infection

**DOI:** 10.1007/s10096-025-05283-6

**Published:** 2025-09-29

**Authors:** Pisut Pongchaikul, Puntabut Warintaksa, Thidathip Wongsurawat, Piroon Jenjaroenpun, Worarat Kruasuwan, Paninee Mongkolsuk, Pornpun Vivithanaporn, Iyarit Thaipisuttikul, Arunee Singhsnaeh, Jakkrit Khamphakul, Suwatcharaporn Hadradchai, Maolee Bhuwapathanapun, Piya Chaemsaithong

**Affiliations:** 1 Ramathibodi Medical School, Chakri Naruebodindra Medical Institute, Faculty of Medicine Ramathibodi Hospital, Mahidol University, Samut Prakan, 10540 Thailand; 2 Integrative Computational BioScience Center, Mahidol University, Nakhon Pathom, 73170 Thailand; 3 Institute of Infection, Veterinary and Ecological Sciences, University of Liverpool, Liverpool, L7 3EA UK; 4 Department of Obstetrics and Gynecology, Faculty of Medicine Ramathibodi Hospital, Mahidol University, Bangkok, Thailand; 5 Division of Medical Bioinformatics, Research Department, Faculty of Medicine Siriraj Hospital, Mahidol University, Bangkok, Thailand; 6 Department of Microbiology, Faculty of Medicine Siriraj Hospital, Mahidol University, Bangkok, Thailand; 7 Department of Pathology, Faculty of Medicine, Ramathibodi Hospital, Mahidol University, Bangkok, Thailand; 8 Program in Translational Medicine, Faculty of Medicine Ramathibodi Hospital, Mahidol University, Bangkok, Thailand

**Keywords:** Acute chorioamnionitis, Acute funisitis, Amniotic fluid, Amniocentesis, Antibiotics, Antibiotic resistance, Ampicillin, Ascending infection, Bacteria, Ceftriaxone, Chorioamnionitis, Commensal bacteria, Comparative genomic, Cultivation, Culture, DNA sequencing, Erythromycin

## Abstract

**Background:**

Intraamniotic infection is a cause of spontaneous preterm labor. *Streptococcus mitis* is a common pathogen identified in intraamniotic infection, with the possible route of hematogenous dissemination from the oral cavity or migration from the vaginal canal. However, there are a few reports on *Streptococcus oralis*, a member of the *S. mitis* group, as a cause of pathogen in intraamniotic infection. We reported herein whole genome sequencing and comparative genomic analysis of *S. oralis* strain RAOG5826 that causes intraamniotic infection.

**Results:**

*Streptococcus mitis* was initially identified from amniotic fluid, vaginal swab, and fetal blood of a patient presenting with preterm prelabor rupture of membranes with intraamniotic infection by the use of conventional microbiological methods (biochemical phenotype, MALDI-ToF, 16 S rRNA). Subsequently, this strain was later identified as *S. oralis* RAOG5826 by whole-genome hybrid sequencing. Genes involved in macrolide and tetracycline resistance, namely *ermB* and *tet(M)*, and mutations in penicillin-binding protein were present in the genome. Moreover, potential virulence genes were predicted and compared with other Streptococcal species.

**Conclusion:**

We reported a comprehensive genomic analysis of *S. oralis*, which causes intraamniotic infection. *S. mitis* was initially identified by conventional microbiological identification. However, whole-genome hybrid sequencing demonstrates *S. oralis* with complete profiles of antimicrobial resistance genes and potential virulence factors. This study highlights the limitations of traditional techniques and underscores the importance of genomic sequencing for accurate diagnosis and tailored antimicrobial treatment. The study also suggests that *S. oralis* may be an underestimated pathogen in intraamniotic infection.

**Supplementary Information:**

The online version contains supplementary material available at 10.1007/s10096-025-05283-6.

## Introduction

Streptococci consist of more than 50 species and are considered one of the largest Genera in the Kingdom of Bacteria. This genus can be divided into three groups (alpha-, beta-, and gamma-hemolytic Streptococcus) based on their hemolytic activity on Sheep’s blood agar [1]. Moreover, they can be classified based on 16 s rRNA gene sequence into seven groups: (1) pyogenic group; (2) anginosus group; (3) mitis group; (4) bovis group; (5) mutans group; (6) salivarius group; and (7) sanguinis group [2–4].

The Mitis group of Streptococci includes most viridans group streptococci (VGS), including *Streptococcus pneumoniae*,* S. mitis*,* S. oralis*,* S. gordonii*,* S. sanguinis*,* S. parasanguinis*,* S. pseudopneumoniae*,* S. infantis*,* S. cristatus*, and *S. tigurinus* [5–7]. The latter is well-known for its virulence and is a major cause of human respiratory tract infection. On the other hand, the former two streptococci in the mitis group are normal flora in the oral cavity, so-called oral streptococci [8]. They can be transiently transferred to the systemic circulation and cause transient bacteremia, which could be the source of infective endocarditis and other systemic infections, including sepsis [9].

*Streptococcus oralis*, a member of *the S. mitis* group, is one of the large, abundant commensals in the human oral cavity, where the species contributes to the early formation of dental plaque [10]. In addition to the oral cavity, *S. oralis* can colonize genitourinary organs, including the vaginal canal [10–12], and it is one of the pathogens causing brain abscess [13], infective endocarditis [10], and urinary tract infection [14]. The identification of Genus Streptococcus based on biochemical characteristics (hemolytic pattern on sheep blood agar, oxidase production, catalase production, bacitracin susceptibility, and optochin susceptibility) provides reliable results for certain species, such as *S. pyogenes*, *S. agalactiae*, and *S. pneumoniae* [2, 3]. However, these tests and molecular methods, including 16 s rRNA gene [15] and matrix-assisted laser desorption/ionization time-of-flight (MALDI-ToF) [16], do not have a capacity for accurate identification beyond major species group level, especially *S. mitis* and *S. oralis*.

Viridans group streptococci, but not *S. oralis*, have been reported in intraamniotic infection [17–23]. This might be due to the limitations of methods for accurately identifying bacteria at the species level. The importance of accurate identification of bacteria has clinical implications for optimizing antibiotics administration to both mother and fetus [21–25].

Our group reported that the *S. oralis* group causes intraamniotic infection, maternal bacteremia, and clinical chorioamnionitis in a patient with preterm prelabour rupture of membranes, and this bacterium might be associated with periodontitis [20]. Species identification was performed using whole-genome sequencing and comparative genomic analysis. We also reported its virulence and antimicrobial resistance genes.

## Materials and methods

### Specimen collection, bacterial identification, and susceptibility test

Clinical specimens, including vaginal swabs, amniotic fluid, fetal blood, and placental swab (space between chorioamniotic membranes), were collected from a patient presenting with preterm prelabor rupture of membranes. Briefly, a vaginal swab was performed at the time of hospital admission. Amniotic fluid was collected by transabdominal amniocentesis in a sterile fashion. Fetal blood was collected in a sterile fashion at the time of Cesarean delivery. After Cesarean delivery, the placental swab was collected at the chorioamniotic space after the chorion and the amniotic membrane were separated using a sterile technique. Vaginal and placental swabs, as well as amniotic fluid and fetal blood, were transported to the clinical microbiology unit for aerobic culture, anaerobic culture, and 16S rRNA gene PCR for bacterial identification using 27F and 1492R primers, followed by Sanger sequencing. The amniotic fluid culture was executed according to the aerobic and anaerobic culture of body fluid. The clinical isolates RAOG5826-1, RAOG5826-2, and RAOG5826-3 were recovered from amniotic fluid, vaginal swab, and fetal cord blood, respectively. There was no bacterial growth from the placental swab.

Antimicrobial susceptibility test was performed using broth microdilution (Sensititre, Thermo Fischer Scientific). Interpretation of antimicrobial susceptibility was performed following the Clinical Laboratory Standard Institute (CLSI) guideline (M100, 35th edition). Penicillin, ampicillin, vancomycin, clindamycin, daptomycin, erythromycin, linezolid, levofloxacin, and tetracycline were used to evaluate antibiotic susceptibility following the interpretation criteria of “*Streptococcus spp.* Viridans group”. The specimen collection and processing were approved by the Institutional Research Board of the Faculty of Medicine, Ramathibodi Hospital, Mahidol University (COA.MURA2021/254 and COA.MURA2025/254).

## DNA extraction and whole genome sequencing

Genomic DNA was extracted from bacterial colonies obtained from a blood agar plate under aerobic conditions. The quantity of the extracted DNA was assessed by a Qubit^®^ 4.0 Fluorometer (Thermo Fisher Scientific, Waltham, MA, USA). Whole genome sequencing was conducted with single-molecule sequencing (Oxford Nanopore Technologies (ONT), London, UK) and with Illumina™ short-read sequencing. Briefly, DNA library preparation was performed by using the Rapid Barcoding Sequencing Kit (SQK-RBK114.24; ONT). A total of 200 ng of genomic DNA was cleaved with the transposase enzyme to produce chemically modified ends, and a barcode was added to each DNA sample, finally ligated with an adapter. The library was loaded into the R10.4.1 flow cell (ONT) and sequenced with the PromethION24 device (ONT) with 48-h sequencing. For Illumina™ short-read sequencing, 150-bp paired-end libraries were prepared with a TruSeq DNA PCR-free Kit and sequenced with the Illumina™ Novaseq sequencer (Illumina Inc., San Diego, CA, USA). This protocol was approved by the Institutional Biosafety Committee of the Faculty of Medicine at Ramathibodi Hospital, Mahidol University (RAMA-IBC 2022–009).

### De novo Assembly and bioinformatics analysis

A hybrid de novo genome assembly method was employed using both long reads and short reads. Quality control and preprocessing were performed prior to the assembly. Initially, adapter trimming and quality filtering of short reads were conducted using Fastp v0.23.2 [26]. The quality of the filtered reads was subsequently assessed with FastQC v0.11.9 (http://www.bioinformatics.babraham.ac.uk/projects/fastqc/). For long reads, raw signals were basecalled and demultiplexed using Dorado v0.5.1 (https://github.com/nanoporetech/dorado) with the SUP model v4.3.0. Adapter trimming was performed using Porechop v0.2.4 (https://github.com/rrwick/Porechop) and Skewer v0.2.2 [27]. Quality evaluation of the long-read data was carried out using NanoPlot v1.38.0. Filtering was conducted with NanoFilt v2.5.0 [28] to retain only reads with a minimum length of 1,000 bases and an average quality score of at least 9. Genome assembly was performed with Unicycler v0.4.8 [29], which integrates both short and long reads for hybrid assembly. The pipeline includes read alignment, error correction, hybrid assembly, circularization, and rotation to generate a high-quality consensus sequence. Gene annotation of the assembled genome was subsequently performed using DFAST v1.2.14 [30], rapid annotation subsystem technology (RAST) [31], and Prokka [32]. DNA sequence-based species identification for three strains was executed based on maximum-likelihood phylogenetic trees constructed from both 16s rRNA gene sequence in NCBI nucleotide BLAST (accessed on 25th July 2025) and whole genome sequence using EZBiocloud [33] (accessed on 25th July 2025). Read mapping was performed by aligning reads obtained from three strains against the complete assembly obtained from amniotic fluid using Snippy (https://github.com/tseeman/snippy).

The prediction of the presence of antibiotic resistance genes and virulence factors was conducted in ABRicate (https://github.com/tseemann/abricate) based on the comprehensive antimicrobial resistance database (CARD) [34] and the virulence factor database [35]. CRISPR-Cas Finder was used to identify the CRISPR array [36]. The genomic comparison analysis was visualized using BRIGS [37]. Newly reported sequenced genomes in this study were deposited in the NCBI GenBank (Biosample: SAMN46323115- SAMN46323117).

## Results

### Isolation of the strains: phenotype

*Streptococcus* strain RAOG5826-1, RAOG5826-2, and RAOG5826-3 were isolated from amniotic fluid, vaginal, and fetal cord blood, respectively. After routine clinical laboratory examination using both biochemical phenotype and matrix-assisted laser desorption/ionization time-of-flight (MALDI-ToF), the strains belonged to the *Streptococcus mitis* group, but the specific species level could not be identified.

### Species identification and Genomic analysis of S. oralis RAOG5826

For the species identification, a nucleotide BLAST search of the 16 s rRNA gene sequence resulted in *Streptococcus sp. Oral taxon 064 strain W10853* (Accession no. CP016207.1) with an identity percentage of 99.87% (Supplementary Table S1). EZBiocloud further identified that the closest neighbour was *S. oralis subsp. oralis* strain RH_1735_08 (Accession no. NCUN01000004) with an average nucleotide identity (ANI) of 95.05%. The complete genome of strain RAOG5826 was obtained from hybrid genome assembly (the combination of short-read and long-read sequences). There were 1,971,701 bp for the genome of strain RAOG5826 **(**Table 1**)**. The RAST annotation predicted a total number of 1,967 features: 1895 CDs and 72 RNAs. Of 1895 CDSs, 569 CDSs (31%) were categorized into subsystems implemented in RAST. The “Amino acid and derivatives” occupied the largest proportion of the genes with 124 CDSs, followed by the “Carbohydrate” and “Nucleotides and nucleosides” with 106 CDSs and 72 CDSs, respectively. Only 39 CDSs were associated with “Virulence, Disease, and Defense.” One intact *Streptococcus mitis* phage SM1 (PHAGE_Strep_SM1_NC_004996) was identified at the position between 137,933 and 191,704. None of the complete CRISPR-Cas was identified.

**Table 1:** Genomic characteristics of *Streptococcus oralis* strain RAOG5826

	*Streptococcus oralis* strain RAOG5826
GenBank Accession no.	SAMN46323115 - SAMN46323117
Assembly program	Unicycler
No. of contig	1
Assembly length (bp)	1,971,701
G-C content (%)	41.4
Annotation software	Prokka
Coding sequences	1855
tRNA	59
rRNA	12
tmRNA	1

**Fig. 1:**
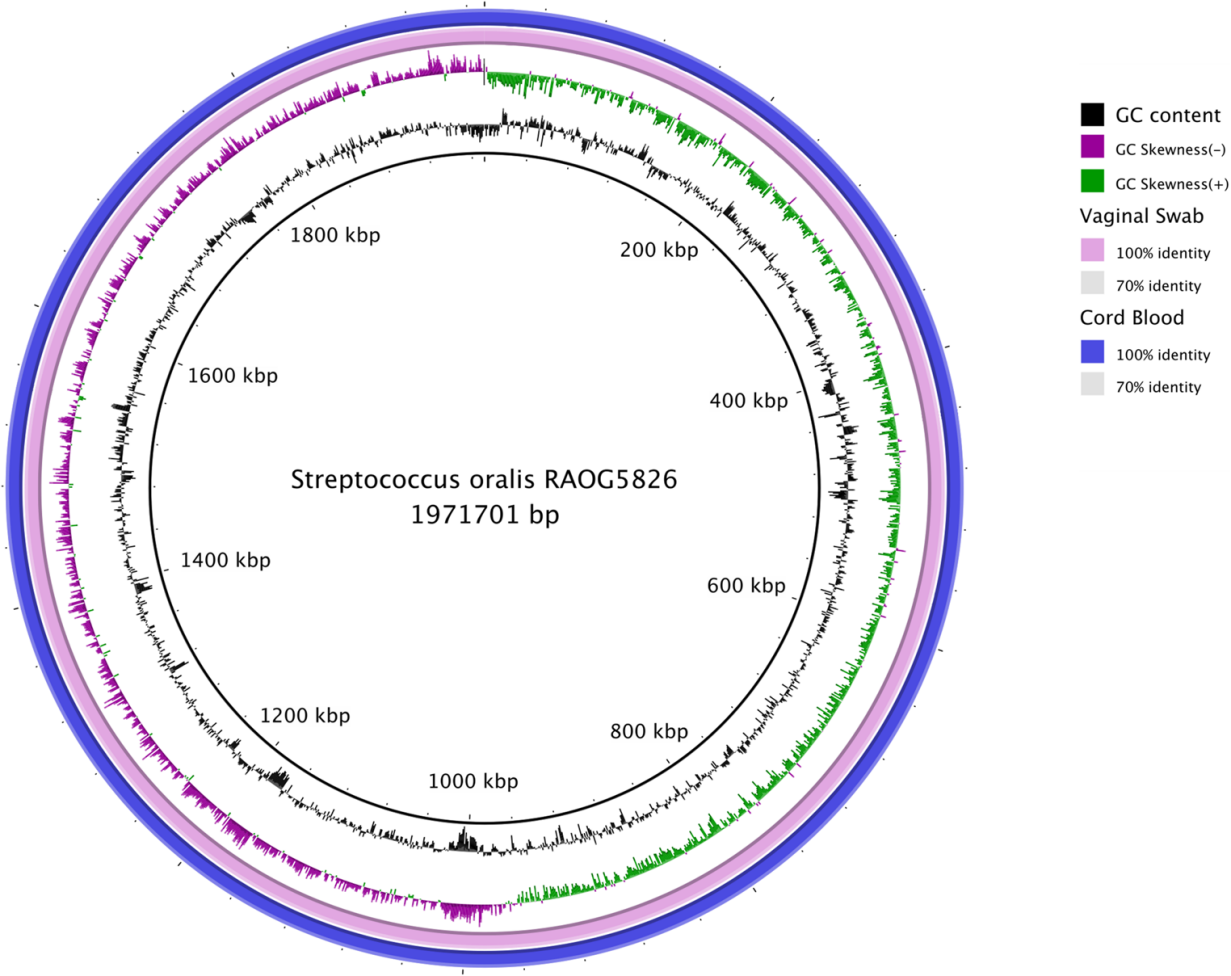
Circular plot of *Streptococcus oralis* RAOG5826 chromosome. Three genomes are obtained from different body sites (amniotic fluid – the innermost ring, vaginal swab – the pink ring, and fetal cord blood – the outermost ring) of the patient with intraamniotic infection

Comparative genomic analysis of the three clinical isolates (RAOG5826-1, RAOG5826-2, and RAOG5826-3) illustrated that the isolates were genetically similar with 7 nucleotide substitution differences (Table 2; Fig. 1), suggesting that these three isolates could be the same organism.

**Table 2:** Nucleotide substitution between the genomes of *S. oralis* strain RAOG5826 collected from different sites (amniotic fluid is used as the reference)

GenBank Accession no.	SAMN46323115 (Reference genome)	SAMN46323116	SAMN46323117
Source	Amniotic fluid	Vaginal swab	Fetal cord blood
Position based on the reference sequence			
293,928	C	C	A
352,291	G	T	G
439,501	G	T	G
439,629	C	C	A
605,041	A	G	G
720,512	C	T	C
826,480	G	G	T
954,866	C	G	G
1,228,343	T	G	G
1,623,293	A	G	G

## Antibiotic susceptibility and antimicrobial resistance genes

The strains exhibited resistant phenotypes to clindamycin, erythromycin, and tetracycline (Table 3). Resfinder identified the *ermB* gene and *tet(M)* gene, acquired antibiotic resistance genes, on the genomes. The occupancy of the *ermB* gene and *tet(M)* gene contributed to clindamycin, erythromycin, and tetracycline resistance of the strain. By investigating genes that are associated with Beta-lactam resistance in more detail, four penicillin-binding proteins (PBP1a, PBP1f, PBP2b, and PBP2x) were identified (Table 3). The analysis of amino acid sequences of penicillin-binding protein identified resistance-related amino acid substitutions, as shown in Table 3.

**Table 3:** Antibiotic susceptibility profile and related genes in strain RAOG5826

Antibiotics	Strain RAOG5826		
	Minimum Inhibitory Concentration (µg/ml)	Interpretation	Gene/mutation identified in this genome
Penicillin	0.25	I	PBP1A: NTGY at positions 574–577 PBP2B: E476G PBP2X: I371T and R384G
Ampicillin	0.25	S	
Vancomycin	0.5	S	-
Clindamycin	> 2	R	ErmB
Daptomycin	≤ 0.5	S	-
Erythromycin	> 4	R	ErmB
Linezolid	1	S	-
Levofloxacin	2	S	-
Tetracycline	8	R	Tet(M)

For interpretation, S = Susceptible; I = Intermediate; R = Resistant

### Virulence genes

The prediction of potential pathogenicity of pathogens was performed by searching the annotated coding sequences of strain RAOG5826 against the virulence factor database (VFDB). By comparing with known alpha-hemolytic *Streptococcus* species, *S. pneumoniae*, *S. sanguinis*, and *S. suis*, the pathogens shared virulence profiles, including genes associated with polysaccharide capsule biosynthesis and fibronectin binding (Table 4). Interestingly, strain RAOG5826 shared several virulence profiles with *S. pneumoniae* (Table 4).

**Table 4:** Comparison of predicted virulence factors of strain RAOG5826 and known *Streptococcus* species

Virulence factor class	Virulence factors	Related genes	RAOG5826 (this study)	S. pneumoniae TIGR4 (NC_003028)	S. sanguinis SK36 (NC_009009)	S. suis 05ZYH33 (NC_009442)
Adherence	Agglutinin receptor		-	-	-	✓
	Antigen I/II (AgI/II) family of oral streptococcal adhesins	*sspA*	✓	-	✓	-
	Cell surface hydrophobicity proteins	*cshA*	-	-	✓	-
	Choline-binding proteins	*cbpD*	✓	✓	-	✓
		*cbpG*	-	✓	-	-
		*lytA*	✓	✓	-	-
		*lytB*	✓	✓	-	-
		*lytC*	-	✓	-	-
		*pce/cbpE*	✓	✓	-	-
		*pspA*	-	✓	-	-
		*pspC/cbpA*	-	✓	-	-
		*cpbA*	-	-	✓	-
	Fibronectin-binding proteins	*fbp54*	-	-	-	✓
		*pavA*	✓	✓	✓	-
	Laminin-binding protein	*lmb*	✓	✓	✓	-
	Muramidase-released protein	*mrp*	-	-	-	✓
	Serine-rich surface glycoproteins	*hsa*	-	-	✓	-
	Sortase A	*srtA*	✓	✓	✓	-
	Streptococcal glucosyltransferases	*gtfD*	✓	-	✓	-
	Streptococcal lipoprotein rotamase A	*slrA*	✓	✓	-	-
	Streptococcal plasmin receptor/GAPDH	*plr/gapA*	✓	✓	✓	-
	rlrA islet	*rrgA*	-	✓	✓	-
		*rrgB*	-	✓	✓	-
		*rrgC*	-	✓	✓	-
		*srtB*	-	✓	-	-
		*srtC*	-	✓	✓	-
		*srtD*	-	✓	-	-
Enzyme	Hyaluronidase	*hylA*	-	-	-	✓
		*hysA*	✓	✓	-	-
	Neuraminidase A	*nanA*	✓	✓	-	-
	Streptococcal enolase	*eno*	✓	✓	✓	-
	Streptococcal phospholipase A2	*slaA*	-	-	-	-
Immune evasion	Capsule biosynthesis		✓	✓	✓	✓
Iron uptake	Pneumococcal iron acquisition	*piaA*	-	✓	-	-
	Pneumococcal iron uptake	*piuA*	✓	✓	-	-
Manganese uptake	Pneumococcal surface antigen A/Metal binding protein SloC	*psaA*	✓	✓	✓	-
Protease	C3-degrading protease	*cppA*	✓	✓	✓	-
	C5a peptidase	*scpA/scpB*	✓	-	-	-
	Extracellular factor	*epf*	-	-	-	✓
	IgA1 protease	*iga*	✓	✓	✓	-
	Serine protease	*htrA/degP*	✓	✓	-	-
	Trigger factor	*tig/ropA*	✓	✓	✓	-
	Zinc metalloproteinase	*zmpB*	-	✓	-	-
		*zmpC*	✓	✓	-	✓
Antiphagocytosis	Capsule (Enterococcus)	*cpsI*	✓	-	-	-

For the presence/absence of genes: “✓” is referred to presence; “-” is referred to absence

## Discussion

### Principal findings of this study

Intraamniotic infection is a leading cause of morbidity and mortality in both pregnant women and neonates worldwide [38–44]. The most common causative pathogens are *Ureaplasma spp*., *Mycoplasma spp.*, and *S. agalactiae* [23, 42, 43]. Advanced DNA sequencing technologies have facilitated the identification of uncommon and unculturable pathogens in intraamniotic infection [23, 45–50]. We herein report the identification of S. oralis using whole genome sequencing, but not from conventional identification methods. These findings support the use of genomic data in clinical medicine.

Strain RAOG5826, causing intraamniotic infection, was initially identified as *S. mitis* using conventional microbiological identification (biochemical phenotype, MALDI-ToF, and 16S rRNA). However, using whole genome sequencing, the strain RAOG5826 was identified as the *S. oralis* group. Although ANI was only 95.05%, which remains ambiguous because the cut-off of ANI for a novel species is 95–96% [51], the analyses of the whole genome sequence using dDDH showed that the strain RAOG5826 genetically belonged to *S. oralis* (dDDH > 70%) [52]. It is well-known that the 16s rRNA sequence and MALDI-ToF are not accurate enough for species identification in particular species, such as the *S. mitis* group, *S. oralis* group [53], and *S. anginosus* group [54]. The additional use of whole genome sequence is required for such species for more precise identification, which has important clinical implications for antimicrobial selection and treatment [24, 50, 53].

Recently, a major concern of viridan group streptococcus infection is related to less antibiotic susceptibility, especially to Beta-lactam, lincosamide, macrolide, and tetracycline. A large study evaluating the viridan group streptococcus in the US from 2010 to 2020 found that the susceptibility rate against penicillin reduced over time, and significant resistance was seen in clindamycin and erythromycin [55]. Strain RAOG5826 showed reduced susceptibility to penicillin and ampicillin, which is supported by the identification of the mutations of concern in PBP in this strain (Table 3). Certain point mutations were associated with reduced susceptibility to beta-lactam [56]. Non-synonymous mutations of PBP affect the physicochemical property of the enzyme, affecting the binding property to the drug.

Resistance to clindamycin, erythromycin, and tetracycline was observed in this strain, as the minimal inhibitory concentration (MIC) value was interpreted as ‘Resistant’ by the CLSI standard. The genomic analysis identified that strain RAOG5826 harbored the *ermB* genes, which are well-known for their macrolide resistance. The *ermB* gene encodes erythromycin-inducible ribosomal methylase that alters the drug molecule binding site on 23S ribosome, resulting in macrolide-lincosamide-streptogramin B (MLSB) resistance [57].

The data about virulence factors of *S. oralis* contributing to intraamniotic infection is limited, as it is an uncommon, identifiable pathogen. A large comparative genomic study showed that there was no specific strain/lineage of *S. mitis* contributing to the pathogenesis of infective endocarditis (IE) [58]. One study in *S. oralis* proposed that C233T of the *nrdM* gene was strongly associated with the invasiveness of the species, but the mutation was a synonymous mutation, and the deletion of the *nrdM* gene did not affect the growth and the fitness of the species [59].

The current pathogenic profile of the species depends on the study reported mainly in IE. In comparison with *S. mitis* and *S. pneumoniae*, *S. oralis* shared several common virulence genes involved in adherence, colonization, and invasion of the pathogen [59]. In this study, we compared the predicted virulence genes of the strain RAOG5826 to well-known *Streptococcus* species, *S. pneumoniae*, *S. sanguinis*, and *S. suis*.

Several genes involved in adherence and colonization have been identified in the genome of strain RAOG5826 **(**Table 3**)**. Previous studies revealed that binding to salivary mucin played an important role in the adhesion step of *S. oralis* infection [60]. Chahal et al. demonstrated that *S. oralis* subsp. *oralis* is bound to both MUC5B and MUC7, facilitated by sortase A, an enzyme that covalently links the Gram-positive cell wall to LPXTG-containing protein. The attachment to mucin pioneers the formation of oral plaque, which is proposed as the early phase of IE. Likewise, the vaginal fluid contains MUC5B and MUC7 [61], which could be adhered to sortase A-harboring *S. oralis*. This may provide the process of colonization and invasion, suggesting the migration into the amniotic cavity.

Pneumococcal adherence and virulence factor A (PavA) are other major factors facilitating the adhesion and invasion into the host by binding to human fibronectin [59]. Lack of expression of the *pavA* gene showed the reduction of adherence and invasion through the epithelial layers in pneumococcus [61], but there is no experimental evidence in *S. oralis*. In addition to PavA, pneumococcal surface adhesion A (PsaA) plays an important role in the adhesion of *S. pneumoniae* to the host cell. The PsaA-mutant pneumococcus adhesion ability was reduced significantly [59]. Neuraminidase A (NanA) is an enzyme that is crucial for platelet binding, which is an important pathogenesis of IE caused by *S. oralis* [62].

Once the bacterial cells can affirm their colonization, invasion is a subsequent step in which bacteria evade host immunity and spread thoroughly inside the host. We identified genes associated with invasion in the strain RAOG5826 **(**Table 3**)**. Immunoglobulin A1 protease, zinc metalloprotease, and autolysin are enzymes that prevent *S. pneumoniae* from host immune cells by degrading host immune components. However, the significance of these enzymes in *S. oralis* infection remains unclear.

### Strengths and limitations

The strength of this study is that clinical specimens were systematically collected from multiple body sites (vaginal swab, amniotic fluid, and fetal blood) of the patient presenting with preterm prelabor rupture of membranes. The comprehensive genomic analysis of *S. oralis* was performed to demonstrate potential antibiotic resistance genes and predicted virulence factors by comparing with other related Streptococcus species. However, the main limitation of this study is that the findings are based on a single case. Future studies are required to confirm our findings in a larger sample size with different types of microorganisms causing intraamniotic infection. In addition, in vitro validation of the virulence factors of *S. oralis* should be further investigated.

## Conclusion

We reported a comprehensive genomic analysis of *S. oralis*, which causes intraamniotic infection. *S. mitis* was initially identified by conventional microbiological identification (biochemical phenotype, MALDI-ToF, 16S rRNA). However, whole-genome hybrid sequencing demonstrates *S. oralis* with complete profiles of antimicrobial resistance genes and potential virulence factors. This study highlights the limitations of traditional techniques and underscores the importance of genomic sequencing for accurate diagnosis and tailored antimicrobial treatment. The study also suggests that *S. oralis* may be an underestimated pathogen in intraamniotic infection.

## Supplementary Information

Below is the link to the electronic supplementary material.

Supplementary Material 1

## Data Availability

Newly reported sequenced genomes in this study were deposited in the NCBI GenBank (Biosample: SAMN46323115- SAMN46323117). All data generated or analyzed are included in this published article. For further information, please contact the corresponding authors.

## Abbreviations

CARD: Comprehensive Antibiotic Resistance Database
CDS: Coding sequences
DNA: Deoxyribonucleic acid
GC: Guanine-Cytosine
IL: Interleukin
Mbp: Million base pairs
MGE: Mobile genetic elements
mL: Milliliter
MLST: Multilocus sequence type
NCBI: National Center for Biotechnology Information
^o^C: Degree Celsius
pg: Picogram
PPROM: Preterm prelabor rupture of the membranes
PROM: Prelabor rupture of the membranes
RAST: Rapid annotation subsystem technology
RNA: Ribonucleic acid
*S. oralis*: *Streptococcus oralis*
ST: Sequence type
tmRNA: Transfer messenger RNA
VFDB: Virulence factor database
VGS: Viridans group Streptococcus
WBC: White blood cell
