# Supplementary material for: Genomic identification and characterization of Streptococcus oralis group that causes intraamniotic infection
Source: Eur J Clin Microbiol Infect Dis. 2025 Sep 29;44(12):3103–13. doi: 10.1007/s10096-025-05283-6 (PMC12753535; doi:10.1007/s10096-025-05283-6)
Supplement: Supplementary file 1 — Supplementary Material 1 [file 10096_2025_5283_MOESM1_ESM.docx]

**Supplementary Table**

**Molecular Characteristics of Streptococcus constellatus Causing Intraamniotic Infection: Pangenome Analysis and Virulence Factor Profiling of the Species Short Title: Streptococcus constellatus and intraamniotic infection**

Supplementary table S1. A table showing the first 10 nucleotide BLAST match of 16s rRNA gene sequence of strain RAOG5826

|  | [Description](https://blast.ncbi.nlm.nih.gov/Blast.cgi) | [Query Cover](https://blast.ncbi.nlm.nih.gov/Blast.cgi?CMD=Get&ADV_VIEW=yes&ADV_VIEW=on&ALIGNDB_BATCH_ID=834238292&ALIGNDB_CGI_HOST=blast.be-md.ncbi.nlm.nih.gov&ALIGNDB_CGI_PATH=/ALIGNDB/alndb_asn.cgi&ALIGNDB_MASTER_ALIAS=SD_ALIGNDB_MASTER&ALIGNDB_MAX_ROWS=100&ALIGNDB_ORDER_CLAUSE=seq_evalue%20asc,aln_id%20asc&ALIGNDB_WHERE_CLAUSE=seq_evalue%20is%20not%20null&ALIGNMENTS=100&ALIGNMENT_VIEW=Pairwise&CONFIG_DESCR=ClustMemNbr,ClustComn,Ds,Sc,Ms,Ts,Cov,Eval,Idnt,AccLen,Acc&DATABASE_SORT=0&DESCRIPTIONS=100&DYNAMIC_FORMAT=on&FORMAT_NUM_ORG=1&FORMAT_OBJECT=Alignment&FORMAT_TYPE=HTML&GET_SEQUENCE=yes&I_THRESH=&LINE_LENGTH=60&MASK_CHAR=2&MASK_COLOR=1&NUM_OVERVIEW=100&PAGE=MegaBlast&QUERY_INDEX=0&QUERY_NUMBER=0&RID=8XBWG3BH013&SHOW_LINKOUT=yes&SHOW_OVERVIEW=yes&STEP_NUMBER=&USE_ALIGNDB=true&ADV_VIEW=on&DISPLAY_SORT=4&HSP_SORT=0) | [E value](https://blast.ncbi.nlm.nih.gov/Blast.cgi?CMD=Get&ADV_VIEW=yes&ADV_VIEW=on&ALIGNDB_BATCH_ID=834238292&ALIGNDB_CGI_HOST=blast.be-md.ncbi.nlm.nih.gov&ALIGNDB_CGI_PATH=/ALIGNDB/alndb_asn.cgi&ALIGNDB_MASTER_ALIAS=SD_ALIGNDB_MASTER&ALIGNDB_MAX_ROWS=100&ALIGNDB_ORDER_CLAUSE=seq_evalue%20asc,aln_id%20asc&ALIGNDB_WHERE_CLAUSE=seq_evalue%20is%20not%20null&ALIGNMENTS=100&ALIGNMENT_VIEW=Pairwise&CONFIG_DESCR=ClustMemNbr,ClustComn,Ds,Sc,Ms,Ts,Cov,Eval,Idnt,AccLen,Acc&DATABASE_SORT=0&DESCRIPTIONS=100&DYNAMIC_FORMAT=on&FORMAT_NUM_ORG=1&FORMAT_OBJECT=Alignment&FORMAT_TYPE=HTML&GET_SEQUENCE=yes&I_THRESH=&LINE_LENGTH=60&MASK_CHAR=2&MASK_COLOR=1&NUM_OVERVIEW=100&PAGE=MegaBlast&QUERY_INDEX=0&QUERY_NUMBER=0&RID=8XBWG3BH013&SHOW_LINKOUT=yes&SHOW_OVERVIEW=yes&STEP_NUMBER=&USE_ALIGNDB=true&ADV_VIEW=on&DISPLAY_SORT=0&HSP_SORT=0) | [Percentage](https://blast.ncbi.nlm.nih.gov/Blast.cgi?CMD=Get&ADV_VIEW=yes&ADV_VIEW=on&ALIGNDB_BATCH_ID=834238292&ALIGNDB_CGI_HOST=blast.be-md.ncbi.nlm.nih.gov&ALIGNDB_CGI_PATH=/ALIGNDB/alndb_asn.cgi&ALIGNDB_MASTER_ALIAS=SD_ALIGNDB_MASTER&ALIGNDB_MAX_ROWS=100&ALIGNDB_ORDER_CLAUSE=seq_evalue%20asc,aln_id%20asc&ALIGNDB_WHERE_CLAUSE=seq_evalue%20is%20not%20null&ALIGNMENTS=100&ALIGNMENT_VIEW=Pairwise&CONFIG_DESCR=ClustMemNbr,ClustComn,Ds,Sc,Ms,Ts,Cov,Eval,Idnt,AccLen,Acc&DATABASE_SORT=0&DESCRIPTIONS=100&DYNAMIC_FORMAT=on&FORMAT_NUM_ORG=1&FORMAT_OBJECT=Alignment&FORMAT_TYPE=HTML&GET_SEQUENCE=yes&I_THRESH=&LINE_LENGTH=60&MASK_CHAR=2&MASK_COLOR=1&NUM_OVERVIEW=100&PAGE=MegaBlast&QUERY_INDEX=0&QUERY_NUMBER=0&RID=8XBWG3BH013&SHOW_LINKOUT=yes&SHOW_OVERVIEW=yes&STEP_NUMBER=&USE_ALIGNDB=true&ADV_VIEW=on&DISPLAY_SORT=3&HSP_SORT=3) identity | matched genome accession |
| --- | --- | --- | --- | --- | --- |
| 1 | [Streptococcus sp. oral taxon 064 strain W10853 sequence](https://blast.ncbi.nlm.nih.gov/Blast.cgi#alnHdr_1043184785) | 100% | 0.00 | 99.87 | [CP016207.1](https://www.ncbi.nlm.nih.gov/nucleotide/CP016207.1?report=genbank&log$=nucltop&blast_rank=1&RID=8XBWG3BH013) |
| 2 | [Streptococcus oralis strain S.MIT/ORALIS-351 chromosome, complete genome](https://blast.ncbi.nlm.nih.gov/Blast.cgi#alnHdr_1140122741) | 100% | 0.00 | 99.81 | [CP019562.1](https://www.ncbi.nlm.nih.gov/nucleotide/CP019562.1?report=genbank&log$=nucltop&blast_rank=2&RID=8XBWG3BH013) |
| 3 | [Streptococcus oralis strain E628 chromosome, complete genome](https://blast.ncbi.nlm.nih.gov/Blast.cgi#alnHdr_2945935328) | 100% | 0.00 | 99.81 | [CP185267.1](https://www.ncbi.nlm.nih.gov/nucleotide/CP185267.1?report=genbank&log$=nucltop&blast_rank=3&RID=8XBWG3BH013) |
| 4 | [Streptococcus oralis strain FDAARGOS_1075 chromosome, complete genome](https://blast.ncbi.nlm.nih.gov/Blast.cgi#alnHdr_1948640724) | 100% | 0.00 | 99.81 | [CP066041.1](https://www.ncbi.nlm.nih.gov/nucleotide/CP066041.1?report=genbank&log$=nucltop&blast_rank=4&RID=8XBWG3BH013) |
| 5 | [Streptococcus oralis strain E664 chromosome, complete genome](https://blast.ncbi.nlm.nih.gov/Blast.cgi#alnHdr_2945929381) | 100% | 0.00 | 99.81 | [CP185270.1](https://www.ncbi.nlm.nih.gov/nucleotide/CP185270.1?report=genbank&log$=nucltop&blast_rank=5&RID=8XBWG3BH013) |
| 6 | [Streptococcus sp. 1643 chromosome, complete genome](https://blast.ncbi.nlm.nih.gov/Blast.cgi#alnHdr_1680453830) | 100% | 0.00 | 99.81 | [CP040231.1](https://www.ncbi.nlm.nih.gov/nucleotide/CP040231.1?report=genbank&log$=nucltop&blast_rank=6&RID=8XBWG3BH013) |
| 7 | [Streptococcus sp. ST14 gene for 16S ribosomal RNA, partial sequence](https://blast.ncbi.nlm.nih.gov/Blast.cgi#alnHdr_2785196113) | 100% | 0.00 | 99.81 | [LC833331.1](https://www.ncbi.nlm.nih.gov/nucleotide/LC833331.1?report=genbank&log$=nucltop&blast_rank=7&RID=8XBWG3BH013) |
| 8 | [Streptococcus oralis strain E684 chromosome, complete genome](https://blast.ncbi.nlm.nih.gov/Blast.cgi#alnHdr_2945918849) | 100% | 0.00 | 99.81 | [CP185274.1](https://www.ncbi.nlm.nih.gov/nucleotide/CP185274.1?report=genbank&log$=nucltop&blast_rank=8&RID=8XBWG3BH013) |
| 9 | [Streptococcus oralis strain CCUG 53468 chromosome, complete genome](https://blast.ncbi.nlm.nih.gov/Blast.cgi#alnHdr_2276948234) | 100% | 0.00 | 99.74 | [CP029257.1](https://www.ncbi.nlm.nih.gov/nucleotide/CP029257.1?report=genbank&log$=nucltop&blast_rank=9&RID=8XBWG3BH013) |
| 10 | [Streptococcus mitis NCTC 12261 chromosome, complete genome](https://blast.ncbi.nlm.nih.gov/Blast.cgi#alnHdr_1611627092) | 100% | 0.00 | 99.74 | [CP028414.1](https://www.ncbi.nlm.nih.gov/nucleotide/CP028414.1?report=genbank&log$=nucltop&blast_rank=10&RID=8XBWG3BH013) |
